# AK-MADDPG-Based Antijamming Strategy Design Method for Frequency Agile Radar

**DOI:** 10.3390/s24113445

**Published:** 2024-05-27

**Authors:** Zhidong Zhu, Xiaoying Deng, Jian Dong, Cheng Feng, Xiongjun Fu

**Affiliations:** 1 Beijing Institute of Technology, Beijing 100081, China; 3120210693@bit.edu.cn (Z.Z.); xydeng@bit.edu.cn (X.D.);; 2 Tangshan Research Institute of BIT, Tangshan 063007, China

**Keywords:** frequency agility, radar, antijamming strategies, multi-agent reinforcement learning

## Abstract

Frequency agility refers to the rapid variation of the carrier frequency of adjacent pulses, which is an effective radar active antijamming method against frequency spot jamming. Variation patterns of traditional pseudo-random frequency hopping methods are susceptible to analysis and decryption, rendering them ineffective against increasingly sophisticated jamming strategies. Although existing reinforcement learning-based methods can adaptively optimize frequency hopping strategies, they are limited in adapting to the diversity and dynamics of jamming strategies, resulting in poor performance in the face of complex unknown jamming strategies. This paper proposes an AK-MADDPG (Adaptive K-th order history-based Multi-Agent Deep Deterministic Policy Gradient) method for designing frequency hopping strategies in frequency agile radar. Signal pulses within a coherent processing interval are treated as agents, learning to optimize their hopping strategies in the case of unknown jamming strategies. Agents dynamically adjust their carrier frequencies to evade jamming and collaborate with others to enhance antijamming efficacy. This approach exploits cooperative relationships among the pulses, providing additional information for optimized frequency hopping strategies. In addition, an adaptive K-th order history method has been introduced into the algorithm to capture long-term dependencies in sequential data. Simulation results demonstrate the superior performance of the proposed method.

## 1. Introduction

Compared to fixed frequency radar, frequency agile (FA) radar exhibits higher resistance to jamming by swiftly adjusting frequency points to avoid jamming bands or dilute jamming power spectral density [1,2]. However, with the continuous advancement of jamming technologies, jammers can analyze the modulation patterns of FA radar, adaptively adjusting jamming strategies to counter frequency agile signals, challenging the antijamming performance of traditional FA radar [3,4,5]. Therefore, it is crucial to conduct research on intelligent antijamming for FA radar systems.

Reinforcement learning (RL) theory has been widely applied to the research of intelligent antijamming decision-making problems in radar. It is a branch of machine learning aimed at enabling agents to take appropriate actions to obtain high rewards [6,7]. The development and maturity of RL-related theories have significantly enhanced radar situational awareness, autonomous learning, and decision-making capabilities [8,9,10,11]. Introducing RL into the radar antijamming field and optimizing radar antijamming strategies through collecting interaction information between radar and jammer can improve the intelligence of radar antijamming decision-making capability [12,13,14].

Traditional RL struggles to handle problems with excessively large state spaces or continuous action spaces. Deep reinforcement learning (DRL) combines deep neural networks with RL, using neural networks to approximate value functions or policy functions to address the shortcomings of traditional RL [15]. For example, Deep Deterministic Policy Gradient (DDPG) is a DRL algorithm based on actor-critic networks that perform well in policy optimization problems [16,17]. The Multi-Agent Deep Deterministic Policy Gradient (MADDPG) algorithm extends the DDPG algorithm to the multi-agent domain, introducing other agents’ actions as additional information to obtain Q-value functions [18].

Further improvements and optimizations are still required for the use of RL and DRL to address the intelligent antijamming problem of FA radar. Ref. [19] employed array beamforming data and jamming status characteristics after pulse compression sensing as model inputs, implementing intelligent updates of the antijamming knowledge base using Q-learning and SARSA algorithms to determine the optimal antijamming strategy based on the knowledge base. Ref. [20] selected 12 antijamming measures using an improved DDPG algorithm, with jamming state changes before and after the implementation of antijamming measures as feedback. Ref. [21] employed the DDPG-MADDPG (Deep Deterministic Policy Gradient and the Multi-Agent Deep Deterministic Policy Gradient) method for adaptive selection of multiple domain antijamming measures, including composite jamming types, using the antijamming improvement factor as feedback. Refs. [19,20,21] all require online recognition of jamming and acquisition of jamming state information to intelligently select antijamming measures. However, the complexity and diversity of jamming signals make the design of online recognition algorithms another challenge. Additionally, mitigating the impact of jamming online recognition on the real-time nature of radar decision-making is also a problem that needs to be addressed.

Ref. [22] utilized the past radar transmission behavior as state input, designing an antijamming strategy for FA radar based on DQN. Ref. [23] proposed a robust antijamming strategy design method by parameterizing the jamming strategy and incorporating jamming strategy perturbations into Wasserstein robust reinforcement learning. The methods presented in [22,23] focus on devising antijamming strategies specifically tailored to known jamming tactics, yet they do not delve into their applicability in scenarios involving unknown jamming tactics.

In complex electronic warfare scenarios, it is often challenging to accurately obtain the jamming status of jammers through real-time online identification. For radar systems, the jamming strategies of jammers are also unknown and dynamically changing. The dynamic nature of jamming strategies is reflected in the fact that jammers choose different jamming strategies when facing different scenarios or at different stages. Therefore, it is necessary to explore the feasibility and effectiveness of intelligent decision-making in complex unknown jamming strategy scenarios. Ref. [24] used radar transmission behavior and signal-to-interference ratio of echo signals as state inputs, optimizing cognitive radar antijamming frequency hopping strategy using two algorithms: Q-learning and Deep Q-Network (DQN). In this study, the jammer model is unknown; thus, the jamming strategy faced by the FA radar is unknown. However, the jamming strategy faced by the radar in the simulation is fixed and single, without considering complex jamming strategies. Ref. [25] employs radar detection probability as the reward function and utilizes proximal policy optimization (PPO) to solve the optimization problem of FA radar antijamming strategies. Additionally, it proposes a unified antijamming strategy learning method based on policy distillation technique to combat multiple jamming strategies. The paper considers the correlation between the target returns, which affects radar detection performance. It designs the state of the agent using the K-th order history method to mitigate partial observability issues, thereby enhancing the optimization performance of the algorithm. Ref. [26], inspired by the successful application of single-agent reinforcement learning in cognitive systems, attempted to apply multi-agent deep reinforcement learning (MDRL) to the antijamming decision-making model of cognitive radar. By incorporating cognitive radar and intelligent jammer into the MDRL framework, based on the decision network of DDPG, the study explores the competition between cognitive radar and intelligent jammer, and overcomes the non-stationarity of the environment using the MDRL framework. This demonstrates the effective role of MDRL theory in optimizing antijamming decision-making of cognitive radar in “cooperation-competition” scenarios. Taking into account the potential cooperative relationship among FA radar signal pulses and the optimization problem of FA radar strategies under unknown jamming strategy scenarios is partially observable, the above research provides valuable references and insights into how FA radar can cope with complex and changing unknown jamming strategies.

To improve the antijamming performance of FA radar in complex unknown jamming strategy scenarios, this paper proposes a frequency hopping strategy optimization method based on AK-MADDPG by modeling and simulating the jamming environment and FA radar. The main contributions of this paper are as follows:

- Constructed simulation models for FA radar and jammers. FA radar can control the transmission frequency of signal pulses emitted within a coherent processing time, while jammers do not intercept radar signals when transmitting signals and employ various jamming strategies. For FA radar, the jamming strategies of jammers are unknown.
- Constructed a Markov decision process for the interaction between radar and jammer and designed various elements of multi-agent reinforcement learning. To address the insufficient research on FA radar hopping strategy optimization based on RL in complex unknown jamming strategies scenarios, this paper proposes an AK-MADDPG method for hopping strategy optimization. This method introduces multiple agents to explore the cooperative relationship among radar signal pulses and uses an adaptive K-th order history method to exploit the long-term dependencies of input data.
- Conducted simulation verification. Under the complex jamming conditions of three mixed jamming strategies, compared with traditional random hopping strategy methods, and classical single-agent reinforcement learning DDPG- and MADDPG-based methods, the proposed method in this paper exhibits better antijamming performance.

## 2. Materials

When studying the game between the FA radar and the frequency spot jammer, understanding the specific principles of both is essential. The following briefly outlines the FA radar signal and jamming echo signal model, along with their element design within a multi-agent reinforcement learning framework.

### 2.1. The Principle of Antijamming for FA Radar

For FA radars, frequency agility can be categorized into inter-pulse frequency agility and inter-group frequency agility. The alteration of radar carrier frequencies renders interception and prediction of the carrier frequency by jamming challenging, thereby enhancing radar’s antijamming capabilities. Inter-pulse frequency agility refers to the inclusion of multiple signal pulses within a coherent processing interval (CPI), with the carrier frequency of each pulse being subject to arbitrary changes. Compared to inter-group frequency agility, inter-pulse frequency agility endows radar with greater resilience against jamming.

The FA radar system, operating with inter-pulse frequency hopping, is capable of effectively countering frequency spot jamming and possesses the ability to reduce the probability of radar signals being intercepted.

**Figure 1:**
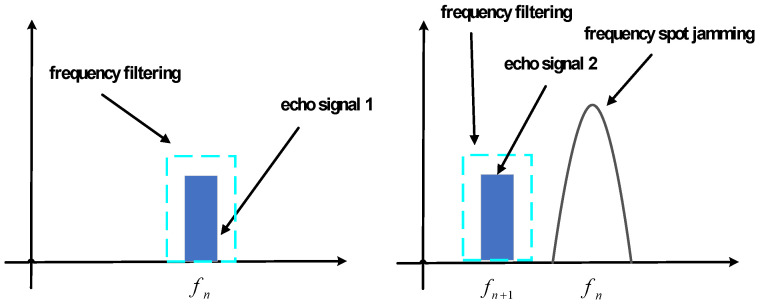
Schematic diagram of inter-pulse FA radar countering frequency spot jamming.

Upon detection of our radar signals by the jammer, high-power noise jamming signals are promptly emitted at the fastest rate to suppress genuine target echoes, rendering the radar unable to detect real targets. This type of jamming can even cause our radar system to overload due to the introduction of excessive high-power false target signals, thereby disrupting normal radar operation. The principle of FA radar countering frequency spot noise suppression jamming is illustrated in the accompanying Figure 1. The carrier frequency utilized for jamming differs from the echo signal’s carrier frequency. Through radio frequency filtering, the energy of frequency spot noise jamming can be significantly suppressed, allowing the FA radar to effectively counteract frequency spot noise jamming.

Due to current limitations, spot jammers cannot simultaneously scan the ultra-wide frequency band when surveying radar signals; rather, they must segment and strategically scan them one by one. Should the radar carrier frequency change between pulses during this process, it would render the jammer unable to intercept all radar signals. Furthermore, the jammer categorizes the detected radar signals based on parameters such as carrier frequency band, pulse repetition frequency, waveform, etc., to determine whether they belong to the same group of radar signals. Consequently, when the radar employs frequency hopping, it severely disrupts the jamming aircraft’s classification of radar signals, greatly reducing the probability of radar signals being intercepted by the jammer.

### 2.2. The Frequency Agile Radar Signal and Jamming Echo Signal Model

**Figure 2:**
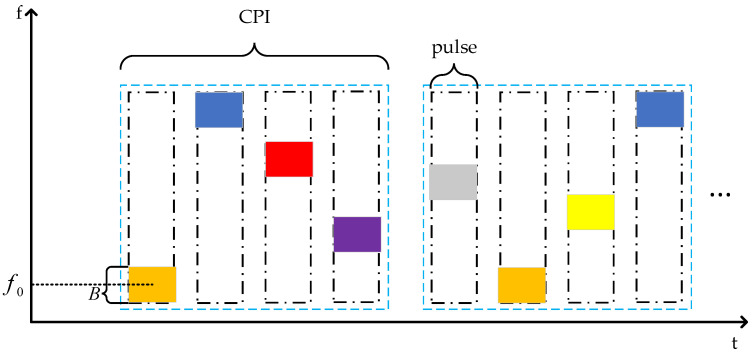
Schematic diagram of inter-pulse frequency agile radar frequency hopping.

Figure 2 illustrates the schematic diagram of inter-pulse frequency agile radar with pseudo-random frequency hopping. Pseudo-random frequency hopping is an extremely flexible frequency hopping method, allowing the radar to transmit signals in other frequency bands to filter out targets and jamming signals when some frequency bands are covered by jammers.

Assuming M linear frequency signals are transmitted by the FA radar within a CPI, with a pulse bandwidth of B, the frequency modulation rate is $\gamma =B/T_{p}$, and $T_{p}$ is the pulse width. The carrier frequency of the m-th pulse is $f_{m}=f_{0}+d_{m}\Delta f$, $d_{m}\in \{0,1,\cdots ,N-1\}$, and N is the maximum number of hopping points, m∈{0,1,⋯,M−1}, and the transmission time of the m-th pulse is $T_{m}$. The m-th signal pulse can be represented as

(1) $$s_{m}\left(t\right)=rect\left(\frac{t-T_{m}}{T_{p}}\right)\exp\left(j\pi \gamma {\left(t-T_{m}\right)}^{2}\right)\exp\left(j2\pi f_{m}\left(t-T_{m}\right)\right)$$

In the equation, t represents the fast time and rect(⋅) represents the rectangular pulse, $rect\left(x\right)=\left\{ \begin{array}{l} 1,\;\left|x/T_{c}\right|\leq 0.5\\ 0,\;\left|x/T_{c}\right|>0.5 \end{array}\right.$.

Assuming the FA radar is dealing with frequency spot noise AM jamming. The received echo signal with jamming by the radar can be expressed as

(2) $$s_{R}(t)=A_{m}rect\left(\frac{t-\tau_{m}}{T_{p}}\right)\exp\left(j\pi \gamma {\left(t-\tau_{m}\right)}^{2}\right)\exp\left(j2\pi f_{m}\left(t-\tau_{m}\right)\right)+n(t)+J(t)$$

where $A_{m}$ and $\tau_{m}$ denote the echo signal amplitude and echo delay of the m-th pulse, respectively, n(t) represents receiver noise, which follows a Gaussian distribution, and J(t) is the frequency spot noise AM jamming.

(3) $$J(t)=[U_{0}+U_{n}(t)]\cos[2\pi f_{j}+\phi ]$$

where $U_{n}(t)$ represents modulated noise with a mean of 0 and variance $\delta^{2}$, which is modeled as Gaussian band-limited white noise. The parameter ϕ follows a uniform distribution and is independent of $U_{n}(t)$. $U_{0}$ denotes the carrier voltage of the jammer. $f_{j}$ represents the center frequency of the jamming signal, which approximates the center frequency of the radar transmission signal targeted by the frequency spot noise AM jamming.

Assuming that the radar cross section (RCS) of the target at this time is δ, the channel gain from the frequency agile radar to the target jammer is $h_{s}$, and the noise power of the environmental clutter is $P_{n}$. Let $f_{m}$ represents the carrier frequency of the m-th pulse within one CPI of the FA radar. The signal-to-interference plus noise ratio (SINR) after pulse compression for the m-th pulse can be expressed as

(4) $$SINR_{m}=\frac{P_{s}h_{s}^{2}\sigma D}{P_{n}+P_{j}h_{s}I\left(f_{j}=f_{m}\right)}$$

where $P_{s}$ represents the transmission power of the frequency-agile radar, and $P_{j}$ denotes the signal power of the frequency spot noise AM jamming. Assuming that the filtering can completely eliminate the jamming signal, I equals 1 if $f_{j}=f_{m}$, otherwise it equals 0. D stands for the time–bandwidth product of the LFM signal.

After pulse compression, followed by coherent processing, in an ideal scenario, the energy accumulation of M echo pulses is $M^{2}$ times that of a single pulse. Since noise samples are independent and zero-mean, the total power of noise is the sum of the powers of individual noise samples. Similarly, the frequency spot noise AM jamming samples entering the receiver, originating from jammers, follow the same statistical distribution, and the total jamming power is the sum of the powers of individual jamming samples. Frequency agility between pulses leads to irregular phase variations in echo signals, preventing direct coherent accumulation. This necessitates phase compensation algorithms and may result in coherent degradation. Consequently, compared to the ideal coherent accumulation scenario, there is a certain loss in SINR after the coherent accumulation of FA radar pulse signals. Therefore, the SINR after coherent accumulation is given by the following:

(5) $$SINR=\frac{M^{2}P_{s}h_{s}^{2}\sigma DL}{MP_{n}+\sum_{m=1}^{M}P_{j}h_{s}I\left(f_{j}^{m}=f_{m}\right)}$$

where $f_{j}^{m}$ denote the jamming frequency corresponding to the timing of the M-th pulse, and L represent the loss factor, which measures the extent of coherent degradation.

### 2.3. The Design of Multi-Agent Reinforcement Learning Elements

**Figure 3:**
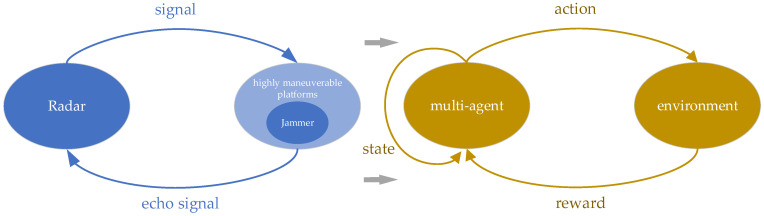
The multi-agent reinforcement learning framework for FA radar and jammer.

As shown in Figure 3, the FA radar and the frequency spot jammer can be regarded as the multi-agent and environment of multi-agent reinforcement learning, respectively. At the time step t, the radar is in state $s_{t}$ and takes action $a_{t}$. The jammer executes jamming actions, causing the environment to transition to the next state $s_{t+1}$, while the radar receives a reward $r_{t}$. This process continues iteratively until the end of the game round.

It is worth noting that in unknown jamming strategy environments, the radar’s input does not include the jammer’s state. The specific design of the reinforcement learning elements is as follows.

#### 2.3.1. Action

Assuming the FA radar contains M signal pulses within one CPI, the radar pulse can be modeled as a multi-agent system comprising M agents. For each signal pulse, its carrier frequency can be selected from a given set of N frequency points.

The FA radar’s action within the t-th CPI can be represented by a vector $a_{t}$ of length 1×M:

(6) $$a_{t}=[a_{0},\cdots ,a_{M-1}]$$

where each element represents the action taken by the corresponding agent, i.e., the carrier frequency of the pulse. The range of values for each element is from 0 to N−1, corresponding to the carrier frequency of pulse from $f_{0}$ to $f_{0}+(N-1)\Delta f$.

The jammer’s actions can also be represented by a 1×M vector $a_{t}^{j}$, which can be encoded according to different situations of the jammer. K can represent the jammer in intermittent observation state, Φ can represent the jammer emitting barrage jamming, k can represent the jammer emitting frequency spot jamming, and k∈{0,1,…,N−1} corresponds to the center frequency $f_{c}+k\Delta f$ of the jamming pulse.

#### 2.3.2. State

In multi-agent reinforcement learning theory, other agents can be regarded as part of the environment for the current agent. Meanwhile, in complex unknown jamming scenarios, radars cannot effectively perceive jamming actions. Since the radar cannot acquire the jamming strategy of the jamming machine, the observation of the environment by the agent only includes the actions taken by other agents. It should be noted that because jamming actions may be related to radar’s historical actions, agents cannot make correct decisions based solely on the previous observation; they need to make decisions based on historical states. In reinforcement learning theory, the historical state $H_{t}$ of the i-th agent at the time T is expressed as follows:

(7) $$H_{T}^{i}=\left\{a_{0}\left[i\right],\cdots ,a_{T-1}\left[i\right],o_{1}^{i},\cdots ,o_{T}^{i}\right\}$$

Here, $o^{i}$ represents the historical actions taken by all agents except the current i-th agent.

(8) $$o^{i}=\left\{a\left[0\right],\cdots ,a\left[j\right],\cdots ,a\left[M-1\right]\right\},j\neq i$$

In unknown environments, if an agent relies solely on observing the actions of other agents to make decisions, then the $H_{t}$ contains all the information that the agent requires. As time progresses, the size of $H_{t}$ also increases, making it impractical for the agent to directly use it as a state. To address this issue, the K-th order history method is employed to approximate the historical states. The K-th order history method is a technique commonly used to handle problems with long-term dependencies. In essence, the K-th order history method approximates $H_{t}$ using the past K observations and actions. Therefore, the state $S_{T}^{i}$ of the i-th agent at the time T can be represented as

(9) $$S_{T}^{i}=\left\{o_{T}^{i},a_{T-1}\left[i\right],o_{T-1}^{i},\cdots ,a_{T-K}\left[i\right]\right\}$$

The states obtained by the K-th order history method are just an approximation of the historical states, thus there may be a significant loss of historical state information in some cases. In unknown environments, due to the lack of prior information, the selection of the K value cannot be based on prior information as a reference, and a poor choice of K value can lead to suboptimal performance in multi-agent reinforcement learning. This paper proposes an adaptive K-th order history method, which can adaptively adjust $S_{T}^{i}$ based on actual feedback. The specific implementation will be explained later in this paper.

#### 2.3.3. Reward

This paper employs the SINR of radar echoes as a metric to characterize the reward obtained by the radar. SINR is an important performance metric in radar systems, indicating the ratio of the target echo signal power to the jamming and noise power received at the receiver. The magnitude of SINR directly impacts the performance and effectiveness of radar systems. A higher SINR implies stronger received signals relative to noise and jamming, thereby enhancing detection capabilities, tracking accuracy, and reducing false alarm rates. Conversely, a lower SINR makes it challenging to distinguish targets from noise and jamming, thereby affecting the detection capabilities and performance of radar systems. The reward for the n-th agent can be expressed as

(10) $$r_{n}=C_{R}*SINR$$

In this context, $C_{R}$ is the reward coefficient. To promote smoothness in reward variation, the logarithm of the SINR is utilized for computation. From the above equation, it can be observed that each agent receives the same reward, which is not only dependent on its own state but also on the states of other agents.

## 3. Methods

In reinforcement learning, the policy followed by an agent when taking actions is denoted as π. The policy represents the mapping relationship from states to actions: π:S→A. Assuming the initial state of the agent is $s_{0}$. The agent transitions to state $s_{1}$ after executing action $a_{0}$ according to policy π and receives rewards $R\left(s_{1},a_{1}\right)$. This continues iteratively, and based on the Markov state transition chain, the total state transition reward value, denoted as $Q_{\pi }(s_{0})$, can be obtained:

(11) $$Q_{\pi }(s_{0})=\sum_{t=0}\gamma^{t}R\left(s_{t},a_{t}\right)$$

Here, γ is the discount factor, used to measure the importance the decision-maker places on future rewards. The discount factor takes values between 0 and 1, where a value closer to 0 indicates more emphasis on immediate rewards, while a value closer to 1 indicates more emphasis on future rewards.

We can evaluate the quality of a policy using the $Q_{\pi }$ function, and we can also assess the value of actions using the state–action value function $Q_{\pi }(s,a)$. It is defined as the expected reward obtained by the agent when executing action a in state s under policy π:

(12) $$Q_{\pi }(s,a)=R(s)+\gamma Q_{\pi }\left(s^{\prime },a^{\prime }\right)$$

where $s^{\prime }$ represents the next state and $a^{\prime }$ represents the action to be taken in the next state under policy π.

The ultimate goal of reinforcement learning is to find an optimal policy ∗π such that the $Q_{\pi }$ function produced by this policy is maximal among all $Q_{\pi }$ functions for all policies:

(13) $$\pi^{*}(a|s)=\{\begin{array}{ll} 1 & \;\mathrm{if}\;a=\underset{a\in A}{\mathrm{argmax}}Q_{*}(s,a)\\ 0 & \;\mathrm{otherwise}\; \end{array}$$

Once the optimal policy ∗π is obtained, the agent can select the optimal action for the current state based on $Q_{*\pi }(s,a)$.

### 3.1. The DDPG Algorithm

**Figure 4:**
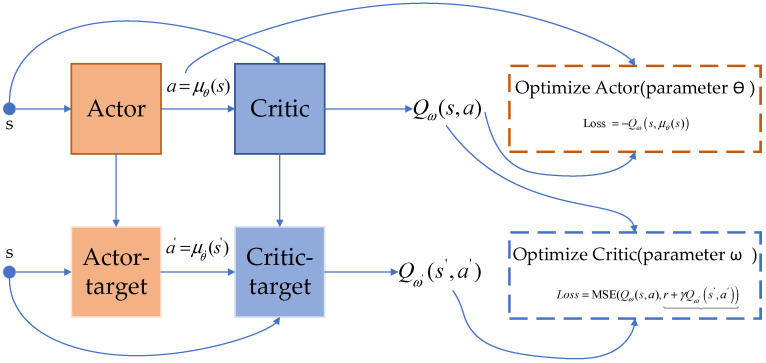
Neural network structure of the agent.

The DDPG algorithm is a method capable of effectively solving or approximating the optimal policy. Its algorithmic structure is illustrated in Figure 4.

As depicted in the diagram, the DDPG algorithm consists of four neural network modules for the agent: the policy network (Actor network), the value network (Critic network), the target policy network (Actor–Target network), and the target value network (Critic–Target network).

The role of the policy network is to output an action a based on the current state s, which can be represented by a function $\mu_{\theta }(s)$. Meanwhile, the value network evaluates the actions outputted by the policy network, corresponding to the state–action value function $Q_{\pi }(s,a)$ mentioned earlier. Initially, the value network, acting as a judge, is unaware of whether the actions output by the policy network are sufficiently good. It needs continuous parameter updates to provide accurate scores. By utilizing the next-step value $Q_{\omega }(s^{\prime },a^{\prime })$ approximated by the target network, along with the actual reward r and the Q-value generated by the value network, Critic’s loss function is constructed to minimize mean squared error.

(14) $$Loss_{critic}=MSE(Q_{\omega }(s,a),r+\gamma Q_{\omega }(s^{\prime },a^{\prime }))$$

This optimization process involves continuously minimizing the loss function. The optimization principle for the policy network is similar. Since the goal of the policy network is to find an action a that maximizes the output value Q of the value network, the method of optimizing the policy network is to maximize the Q-value outputted by the value network. Thus, a loss function can be constructed as follows:

(15) $$Loss_{actor}=-Q_{\omega }(s,\mu_{\theta }(s))$$

The target networks are updated periodically through soft updates, by copying the network parameters of the value network and the policy network at regular intervals and weighing them into the target network parameters by a certain coefficient. The purpose of the target networks is to reduce training instability and improve the algorithm’s performance. The DDPG algorithm solves for the optimal policy by simultaneously optimizing the policy network and the value network.

### 3.2. The Process of the AK-MADDPG Algorithm

For FA radar, effective antijamming often requires cooperation among pulses within a single CPI, indicating a cooperative relationship among pulses. Traditional single-agent systems have struggled to exploit these interdependencies effectively. Moreover, multi-agent reinforcement learning has demonstrated outstanding performance in “cooperative-competitive” scenarios, making it a viable approach for optimizing frequency hopping strategies in FA radar.

The DDPG algorithm has found wide application in the single-agent domain, and its extension to the multi-agent domain yields the MADDPG algorithm. In the MADDPG algorithm, each agent in the multi-agent system has neural networks similar to those in the DDPG algorithm, with each agent possessing its own policy and value networks. Unlike the DDPG algorithm, the MADDPG algorithm introduces actions from other agents as additional information to obtain Q-value functions, where the input to the value network includes not only the current state of the agent but also the states of other agents. This additional input helps agents explore the environment while learning the actions of other agents, prompting them to collaborate to cope with complex unknown external environments.

**Figure 5:**
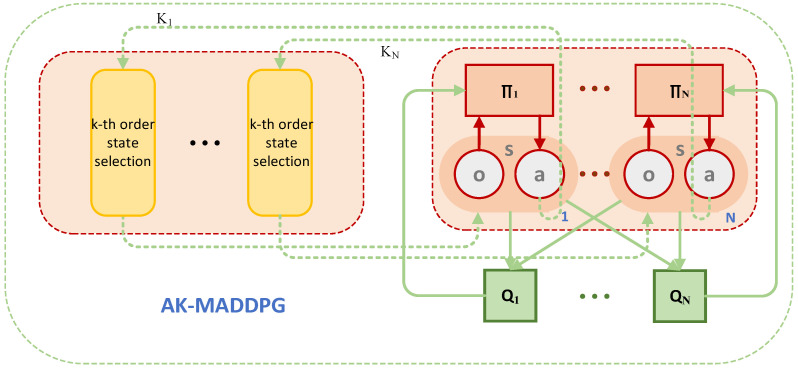
AK-MADDPG algorithm framework.

Given that the actions of jammers may be related to the radar’s historical actions, agents in multi-agent reinforcement learning may struggle to make correct decisions based solely on the previous observation. Therefore, this study improves the MADDPG algorithm by using an adaptive K-th order history method to enhance the input states, enabling them to contain the information necessary for radar decision-making. The improved AK-MADDPG algorithm’s schematic diagram is illustrated in Figure 5.

**Figure 6:**
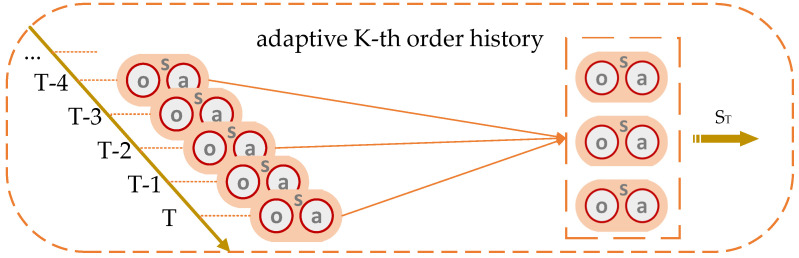
Adaptive K-th order history diagram.

The K-th order history method approximates historical states by considering the past K states approaching the current time. However, the crucial information required for agent decision-making may not necessarily be contained within these states, potentially resulting in suboptimal reinforcement learning outcomes for agents. To address this issue, we propose an adaptive K-th order history method. The principle of the adaptive K-th order history involves adding K actions related to K-th order history into the action space of the agent. The values of these actions determine which past states should be aggregated when approximating history. For instance, as illustrated in Figure 6, at time T, when K = 3, the values of these actions are [0, 2, 4]. Consequently, the observation and action at times T, T-2, and T-4 are combined, rather than simply considering the three adjacent observations and actions at the current time. Through the reinforcement learning process, the agent gradually learns to select the optimal K past states to approximate historical states. This indicates that as the reinforcement learning process progresses, $S_{T}^{i}$ will incorporate more information essential for the agents. It functions akin to a self-attention mechanism, allowing the agent to focus on important information while being resource-efficient and simple to implement.

One of the primary advantages of introducing the adaptive K-th order history method is enhancing the agents’ understanding of the long-term evolution of the environment. By considering the historical states, agents can better capture long-term dependencies in actions and the environment, thereby improving decision stability and reliability. The AK-MADDPG algorithm procedure follows the following procedure (See Algorithm 1):
**Algorithm 1** The AK-MADDPG algorithm procedure1: **Initialize** the network parameters for each agent i:
$\theta_{i}^{Q}$, $\theta_{i}^{\pi }$
2: **for** training epochs from 1 to N **do**3:  Randomly initialize the state s for each agent4:  **for** each timestep T from 0 to the end of a training epochs **do**5:    Select action $a_{i}$ based on the current policy π, state s for each agent i
6:    Execute actions $a_{T}=\left[a_{0},\cdots ,a_{M-1}\right]$, and receive rewards r
7:    State transfer to $s^{\prime }$
8:    Store data $\left(s,a_{i},r,s^{\prime }\right)$ in the buffer $D^{i}$
9:    Update the states: $s\leftarrow s^{\prime }$
10:     **for** each agent i from 1 to M **do**11:      **if** the experience data reaches a trainable amount **do**12:       Extract a set of data from the experience buffer $D^{i}$13:       Compute the loss according to the loss functions14:       Update the network parameters $\theta_{i}^{Q}$, $\theta_{i}^{\pi }$
15:      **end if**16:     **end for**17:   **end for**18: **end for**

## 4. Discussion

This section validates the optimization effectiveness of the frequency hopping strategy based on AK-MADDPG through the simulation experiments.

**Figure 7:**
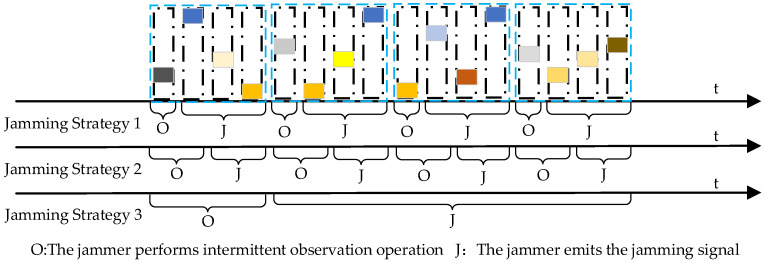
Schematic diagram of the three different jamming strategies.

To validate the effectiveness of the proposed method, we assume that the jammer can adopt three different jamming strategies. As depicted in Figure 7, the distinctions among the three jamming strategies primarily lie in the duration of intermittent observation and the timing of emitting jamming signals. It should be noted that these three strategies belong to basic strategies; the jammer can achieve more complex strategies by dynamically adjusting different jamming strategies over a long game time. The descriptions of the three basic jamming strategies are as follows:

- Jamming Strategy 1: As illustrated in the figure, the intermittent observation time of Jamming Strategy 1 equals the duration of a signal pulse. After intercepting the pulse, the jammer will emit a frequency spot jamming signal.
- Jamming Strategy 2: As shown in the figure, the intermittent observation time of Jamming Strategy 2 equals the duration of two radar pulses, slightly longer than Jamming Strategy 1. Unlike Jamming Strategy 1, to avoid being deceived by the radar, the jamming device will skip the first of the intercepted two pulses, and the center frequency of its frequency spot jamming corresponds to the carrier frequency of the second pulse.
- Jamming Strategy 3: As depicted in the figure, the intermittent observation time of the jammer equals the duration of one CPI, which means the jammer will continuously intercept pulses for some time and use them as a basis for emitting jamming signals thereafter. To avoid being deceived by the radar, the same jammer will skip the first intercepted pulse and then compare the frequencies of the remaining pulses. If the frequencies of the remaining pulses are consistent, the jammer will emit frequency spot jamming with the same center frequency as the intercepted pulse’s carrier frequency. If the remaining pulses correspond to two different frequencies, the jammer will alternately emit frequency spot jamming corresponding to these two different frequencies. If the remaining pulses correspond to more than two different frequencies, the jammer will emit barrage jamming. It is assumed that the duration of jamming lasts for 4 CPIs.

The radar simulation parameters are set as shown in Table 1:

We employ observations and actions from the past K=2 pulses to estimate the agent’s historical observations and behaviors.

The algorithm parameters are configured as shown in Table 2:

In the simulation, it is assumed that the FA radar uses the first pulse within each CPI as a deceptive pulse to induce the jammer to jam on the wrong frequency band. During the coherent processing, the FA radar will exclude the deceptive pulse and process only the remaining pulses. In the cooperative scenario, the reward in multi-agent reinforcement learning is the sum of rewards for all agents. However, since the deceptive pulse does not affect the coherent processing outcome of the FA radar, the reward for this agent is ignored.

**Figure 8:**
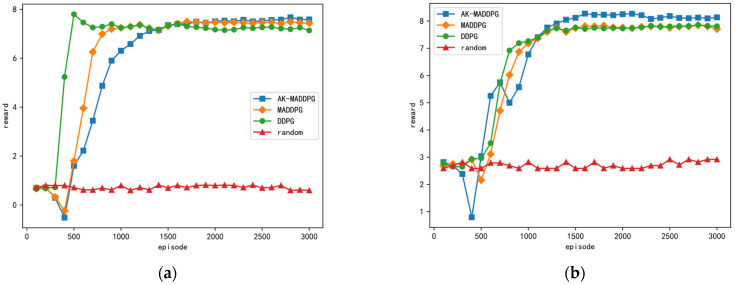

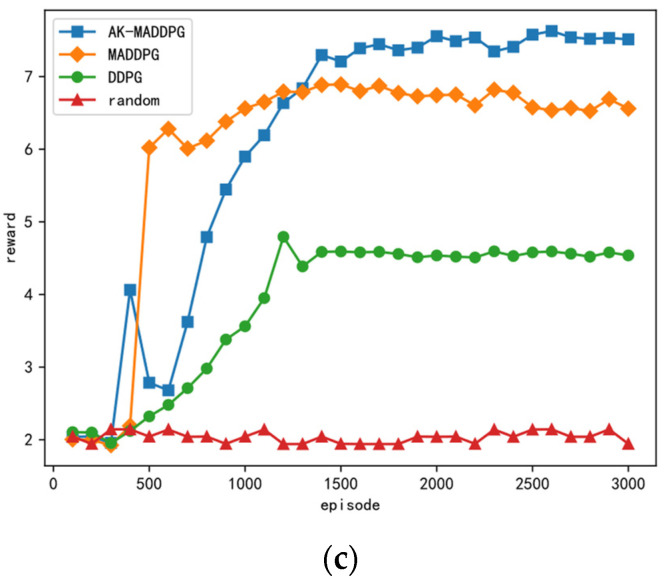
The figures illustrate the reward curves for combating three different jamming strategies using four methods. (**a**), (**b**), and (**c**) depict the reward curves for countering jamming strategies 1, 2, and 3, respectively.

The reward curves for combating the three different jamming strategies using the method proposed in this paper and three other methods are shown in Figure 8. The x-axis represents the number of training epochs, while the y-axis represents the total reward obtained during a single training epoch. It can be observed from the figure that all three DRL-based frequency hopping strategy design methods outperform the random hopping method, demonstrating their effectiveness in learning effective antijamming strategies. Because jamming strategies 1 and 2 are relatively simple, even in the absence of prior knowledge about the jamming strategy, the optimization results of the three DRL-based antijamming strategies can approach the theoretically optimal strategy. As shown in Figure 8c, the performance of the proposed method is significantly better than the other three methods, particularly when dealing with the more complex Jamming Strategy 3. Additionally, the performance of the MADDPG algorithm surpasses that of the DDPG algorithm, highlighting the advantage of multi-agent systems in handling complex scenarios. It is noteworthy that during the first 256 epochs, the performance of the three DRL-based methods is similar to that of the random hopping method because during this period, the algorithm only stores data in the experience buffer without updating the network parameters.

**Figure 9:**
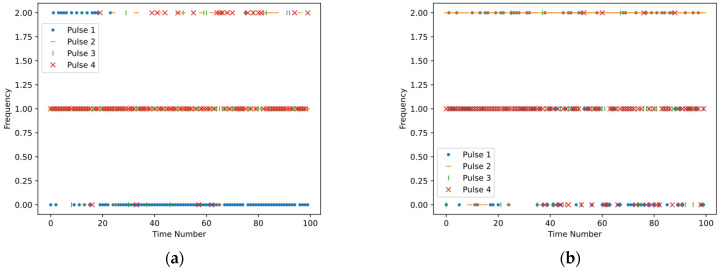

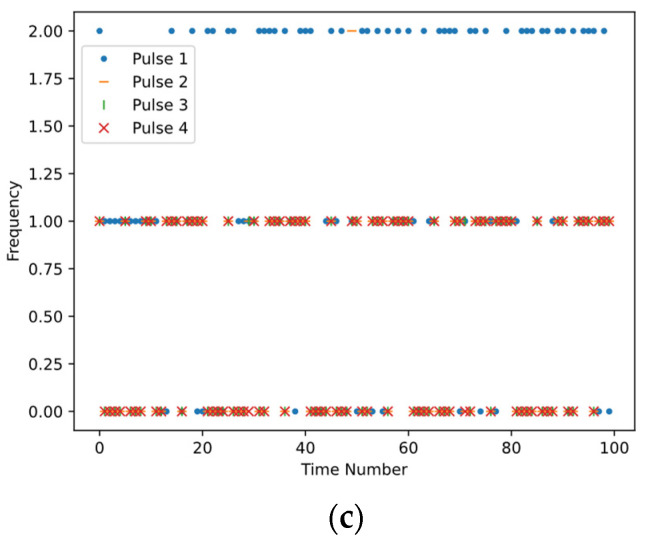
The figures depict the antijamming strategies learned by the method proposed in this paper against three different jamming strategies. (**a**), (**b**), and (**c**) represent the antijamming strategies learned when countering jamming strategies 1, 2, and 3, respectively.

The antijamming strategies learned by the method proposed in this paper against the three jamming strategies are shown in Figure 9. The x-axis represents time, while the y-axis represents the carrier frequency of the pulses. As shown in Figure 9a, most of the frequencies of pulse 1 are $f_{0}$. Since pulse 1 is intercepted by the jammer, other pulses will avoid $f_{0}$ and mostly concentrate around $f_{1}$. Similarly, in Figure 9b, most of the frequencies of pulse 2 are $f_{2}$, and other pulses avoid the frequencies of pulse 2, mostly concentrating around $f_{1}$. As pulse 1 serves as a deceptive pulse and contributes nothing to coherent accumulation, the carrier frequencies are evenly distributed among the three frequencies. In Figure 9c, to counteract Jamming Strategy 3, the carrier frequencies of pulses 2, 3, and 4 tend to be consistent, alternating between $f_{0}$ and $f_{1}$.

**Figure 10:**
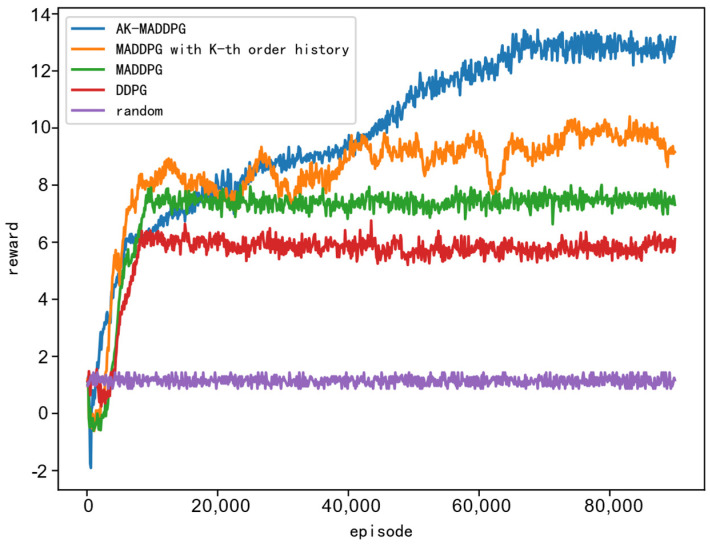
The reward curves under the scenario of mixed jamming strategies.

Building upon the three fundamental jamming strategies, we construct more intricate jamming strategy scenarios through combinations. We assume that the jammer initially employs Jamming Strategy 1, switching to Jamming Strategy 2 upon detecting deceptive pulses, and ultimately adopting Jamming Strategy 3 upon identifying the radar signal as frequency agile. To streamline the simulation, Jamming Strategy 1 and Jamming Strategy 2 are maintained for 20 gaming rounds each, followed by exclusive utilization of Jamming Strategy 3 thereafter. To verify the convergence of the algorithm as the number of pulses increases, the number of pulses within one CPI and available frequency points for the radar are increased. The number of pulses is increased to 8, and the available frequency points are increased to 7. Additionally, to allow for longer convergence time for the algorithms, we extend the training rounds to 90,000. The simulation results are presented in the following Figure 10.

Figure 10 displays the reward curves under the scenario of complex unknown jamming strategies, based on the method proposed in this paper and four other methods. The x-axis represents the number of training epochs, while the y-axis represents the total reward obtained within a single training epoch. From Figure 10, it can be observed that the performance of the random frequency hopping method remains the poorest. The performance of multi-agent reinforcement learning methods surpasses that of single-agent reinforcement learning methods. Moreover, the performance of the method proposed in this paper is significantly better than that of the methods based on MADDPG with K-th order history and MADDPG alone. This indicates the clear advantage of the AK-MADDPG algorithm proposed in this paper when facing scenarios with complex unknown jamming strategies.

## 5. Conclusions

In the context of a single jamming strategy scenario, reinforcement learning-based radar antijamming decision-making methods often deviate from the practical adversarial gaming environment of complex and unknown jamming strategies, thereby limiting their application in practical electronic warfare. To address this issue, this paper proposes a radar frequency agile antijamming strategy optimization method based on multi-agent reinforcement learning, delving into the optimization problem of antijamming strategies for frequency agile radars in complex and unknown jamming strategy scenarios.

In complex and unknown jamming strategy scenarios, transforming the original single-agent problem into a multi-agent problem yields better results. This is because multi-agent reinforcement learning introduces additional information during the training process, enabling better learning and optimization of behavior in complex and unknown environments. Additionally, this paper improves the MADDPG algorithm used by introducing an adaptive K-th order history method to exploit long-term dependencies in input data sequences. Simulation results demonstrate the effectiveness and superiority of the proposed method.

Although the proposed method outperforms other methods when facing complex and unknown jamming strategies, it still falls short of achieving the theoretically optimal antijamming effect, indicating that there is still room for improvement in the algorithm. Moreover, to simplify the analysis, the number of pulses and frequency points in the simulation is set relatively low, which may not fully reflect the actual optimization of frequency agile radar hopping strategies. These aspects will be further refined and improved in future research.

## Figures and Tables

**Table 1:** Radar simulation parameter setting.

Parameter	Value
radar power $P_{s}$	1000 W
jammer power $P_{j}$	80,000 W
noise power $P_{n}$	10 W
time–bandwidth product D	50
initial carrier frequency $f_{0}$	3 GHz
Target’s RCS δ	1 m^2^
number of pulses in one CPI	4
number of available frequency points for the radar	3
channel gain $h_{s}$	0.1

**Table 2:** The algorithm parameters setting.

Parameter	Value
number of training epochs $e_{num}$	3000
number of times the network is updated per epoch $N_{update}$	100
learning rate of the Actor network	1 × 10^−3^
learning rate of the Critic network	1 × 10^−3^
discount factor γ	0.95

## Data Availability

The original contributions presented in the study are included in the article, further inquiries can be directed to the corresponding authors.
